# Machine learning for manually-measured water quality prediction in fish farming

**DOI:** 10.1371/journal.pone.0256380

**Published:** 2021-08-18

**Authors:** Andres Felipe Zambrano, Luis Felipe Giraldo, Julian Quimbayo, Brayan Medina, Eduardo Castillo

**Affiliations:** 1 Department of Electrical and Electronic Engineering, Universidad de los Andes, Bogota, Colombia; 2 Department of Systems Engineering, Corporación Universitaria del Huila, Neiva, Colombia; 3 Department of Veterinary Medicine, Corporación Universitaria del Huila, Neiva, Colombia; Ton Duc Thang University, VIET NAM

## Abstract

Monitoring variables such as dissolved oxygen, pH, and pond temperature is a key aspect of high-quality fish farming. Machine learning (ML) techniques have been proposed to model the dynamics of such variables to improve the fish farmer’s decision-making. Most of the research on ML in aquaculture has focused on scenarios where devices for real-time data acquisition, storage, and remote monitoring are available, making it easy to develop accurate ML techniques. However, fish farmers do not necessarily have access to such devices. Many of them prefer to use equipment to manually measure these variables limiting the amount of available data to process. In this work, we study the use of random forests, multivariate linear regression, and artificial neural networks in scenarios with limited amount of measurements to analyze data from water-quality variables that are commonly measured in fish farming. We propose a methodology to build models in two scenarios: i) estimation of unobserved variables based on the observed ones, and ii) forecasting when a low amount of data is available for training. We show that random forests can be used to forecast dissolved oxygen, pond temperature, pH, ammonia, and ammonium when the water pond variables are measured only twice per day. Moreover, we showed that these prediction models can be implemented on a mobile-based information system and run in an average smartphone that fish farmers can afford.

## Introduction

Monitoring water quality is an important task in fish farming [1, 2]. For example, according to several studies, it is necessary to measure dissolved oxygen to avoid extreme values of this variable that can cause severe damage to fishes such as anoxia, hypoxia, and hyperoxia [3, 4]. The temperature and pH of the water pond also have to be monitored to determine the balance between toxic and non-toxic nitrogen compounds such as ammonia and ammonium [5]. The whole nitrogen cycle should be monitored to recognize an excessive feeding of the fishes and avoid toxic conditions that can harm their health and the production quality of the farm [5]. In recent years, machine learning (ML) has become an important tool to analyze data acquired by those monitoring networks and model the dynamics of the water pond. It typically allows fish farmers to improve their decision-making and maximize their production [6]. Typically, machine learning solutions are based on datasets that are constructed using acquisition devices for the automatic collection of a large amount of data with hourly or minute-based time resolutions. This collection process facilitates the development of accurate ML models to estimate variables such as nitrites and ammonia, and forecast variables such as dissolved oxygen, pond temperature, and pH. However, this is not the case for fish farmers who prefer or are restricted to manually collect one or two daily measurements per variable. For this reason, there is a need to study both variable estimation and forecasting in scenarios that are restricted to one or two measurements per day. In this work, we focus on this situation. Our contribution in this study is an exploratory analysis of the prediction task in the presence of low amount of data collected at very low sampling frequencies for different variables that are commonly measured in fish farming. We focus on two main scenarios: i) estimation of unobserved variables based on the observation of other variables (we refer to this process as variable estimation throughout the paper), and ii) forecasting the behavior of variables based on their previously observed dynamics (we refer to this process as variable forecasting).

We analyze information including pH, dissolved oxygen, temperature, nitrogen compounds, electric conductivity, alkalinity, and meteorological data [7, 8], along with ML techniques that deal with small datasets. It is known that nitrogen compounds, electric conductivity, and alkalinity are correlated with the toxicity of the water pond, the presence of harmful ions, or the impact of pH on the water quality of the pond [5, 9]. Hence, we not only study the accuracy of the models but also the importance of the variables to conduct the estimation and forecasting process. The importance of some of these variables has been analyzed in terms of production [10], but, as far as we know, this has not been studied before for estimation and forecasting of water quality variables in fish farming scenarios. This is particularly helpful to determine which variables have to be measured and which can be avoided to reduce measurement costs. In this way, the costs of measuring can be reduced while having better decision making based on water quality prediction. Additionally, we study the feasibility of the implementation of an information system that uses the proposed models for estimation and forecasting that can be used by fish farmers.

This paper is organized as follows. First, we describe the data collection process in the selected water pond for fish farming and the performance indicator used to evaluate the estimation and forecasting models. Then, we introduce the methodology used to estimate the state of water-quality parameters based on measurements of other variables, and to variable forecasting. Here, we incorporate satellite data into the dataset and provide a comparison between random forests, linear regression, and artificial neural networks for estimation and forecasting. Afterward, we use these models to evaluate the feasibility of the implementation of a decision-making advisor tool for fish farmers. Finally, we finish this paper with a discussion about the usefulness of the methodology and tools developed in this work.

### Related work

Many studies have focused on forecasting dissolved oxygen, which is considered one of the most important variables to guarantee the minimum levels of water quality required for fish farming [7, 8, 11–14]. For example, the work in [11] used multivariate linear regression (LR) and artificial neural networks (ANN) to forecast dissolved oxygen using variables such as pH, temperature, electrical conductivity, and surface runoff. The study in [12] extended the work in [11] to compare ANN and LR with support vector machines (SVM) to forecast dissolved oxygen in a scenario where measurements were collected every 15 minutes. It was shown that all competing models can be accurate for forecasting. However, these models present a significant decrease in the accuracy to forecast dissolved oxygen in a long-term horizon and require a high number of previous observations [12]. To improve the variable prediction accuracy in long-term horizons, several types of deep neural networks have been implemented. For example, the work in [7] implemented decision trees to select the most important features to predict dissolved oxygen, and the selected features were used to train a long-short term memory neural network model (LSTM) to forecast this variable in long-term horizons. Those features are selected from diverse automated monitoring systems and weather stations including more than 10 different types of measurements. Currently, neural network-type models, such as LTSM and deep belief networks [13] are considered an appropriate solution to solve the forecasting problem in long-term horizons [14, 15]. Also, forecasting of other water-quality related variables has been studied, not as much as dissolved oxygen though. For example, temperature and pH have been predicted using linear and non-linear models [16] applying mean reversion in state-space models to increase the accuracy of the forecasting. In general, promising results have been obtained for short-term and long-term forecasting of water-quality variables in fish farming scenarios when these ML methods are applied to measurements collected at sampling times of few minutes, such as 15 minutes, which is the most common case for these studies [12, 16].

Another approach that has been studied is the estimation of unobserved variables. This scenario is particularly useful when a variable cannot be measured at a given time, and estimations have to be obtained based on the information provided by other variables. Although this approach has not been studied as much as the forecasting one, reactive phosphorus, nitrites and ammonia have been estimated using other variables measured with automated in-situ instrumentation [17]. Random forests models have shown high accuracy results for the estimation scenario when measurements have a time resolution of minutes [17]. However, as far as we know, this approach has not been studied when the frequency of data collection is low.

## Materials and methods

**Fig 1:**
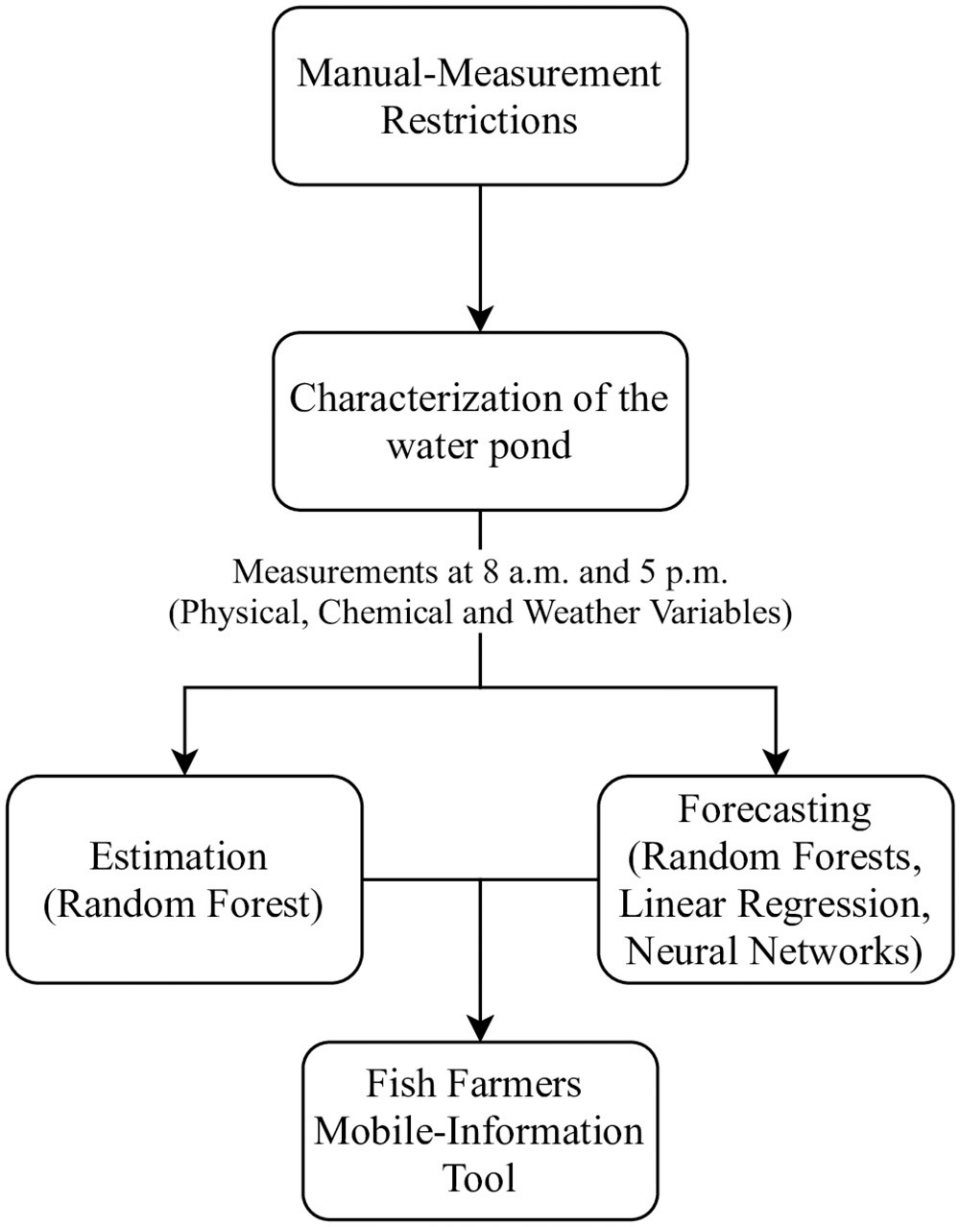
Process followed in this study.

Fig 1 shows the experimental process that we followed in this study. First, to collect the training data, we conducted a preliminary experiment to determine the most suitable hours to measure the target variables. Once the data was collected, we trained, tested, and analyzed the machine learning models for variable estimation and forecasting. Using the resultant models, we evaluate the feasibility of the implementation of a mobile information tool for prediction in scenarios where measurements are manually collected. Following, we describe the data collection task, the used machine learning model, and the performance indicators for prediction.

### Data collection

The first step of this study is to determine the hours at which the measurements are collected. Some studies suggest that measurements should be collected at dawn and sunset because of the respiration and photosynthesis cycles of the algae in ponds [3, 5]. In this study, we measured environmental and pond temperature, dissolved oxygen, pH, electric conductivity, alkalinity, and mass concentration of ammonia, ammonium, and nitrites every three hours for two days to recognize maximum and minimum values of each variable, the hour when these extreme values happen and their tendencies. Based on this information, we can select the hours to measure water-quality variables, restricted to 2 measurements per day.

**Fig 2:**
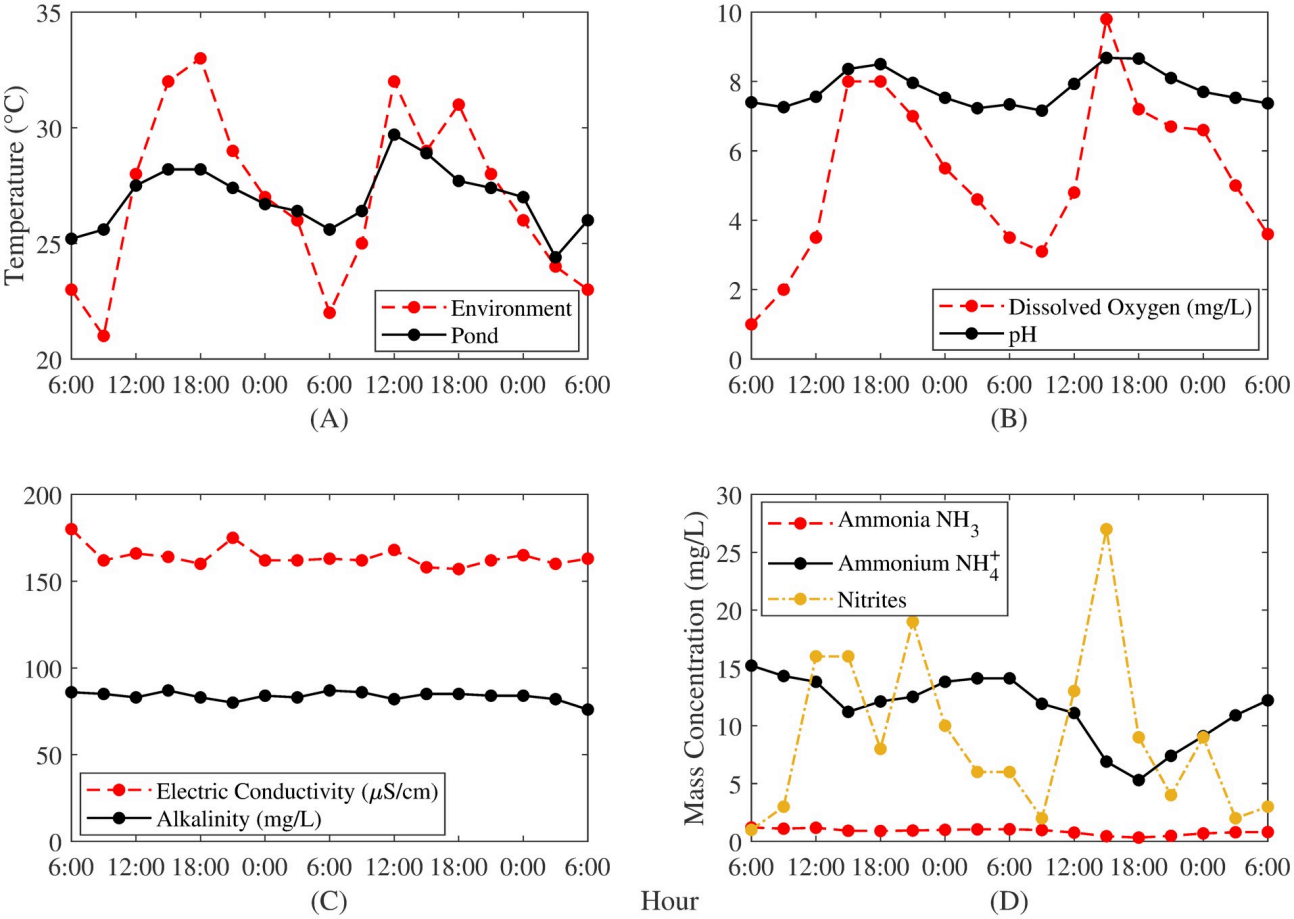
Location of the water pond. Geographic coordinates are 2.796162372495068, -75.29639700197488.

The water pond that is studied here is located at coordinates (2.796162372495068, -75.29639700197488), close to the equator. Fig 2 shows the geographic location of the pond. This pond is made by a small dam over a river with two fish cages, allowing farmers to have a natural water replacement. Fish are fed twice in the morning and one or two times in the afternoon, which can hinder noticing an impact on nitrites and ammonia values using a time periodicity of three hours. However, the impact of feeding is not as important as the relationship between temperature and pH on water ponds with natural replacement, which is observed here.

**Fig 3:**
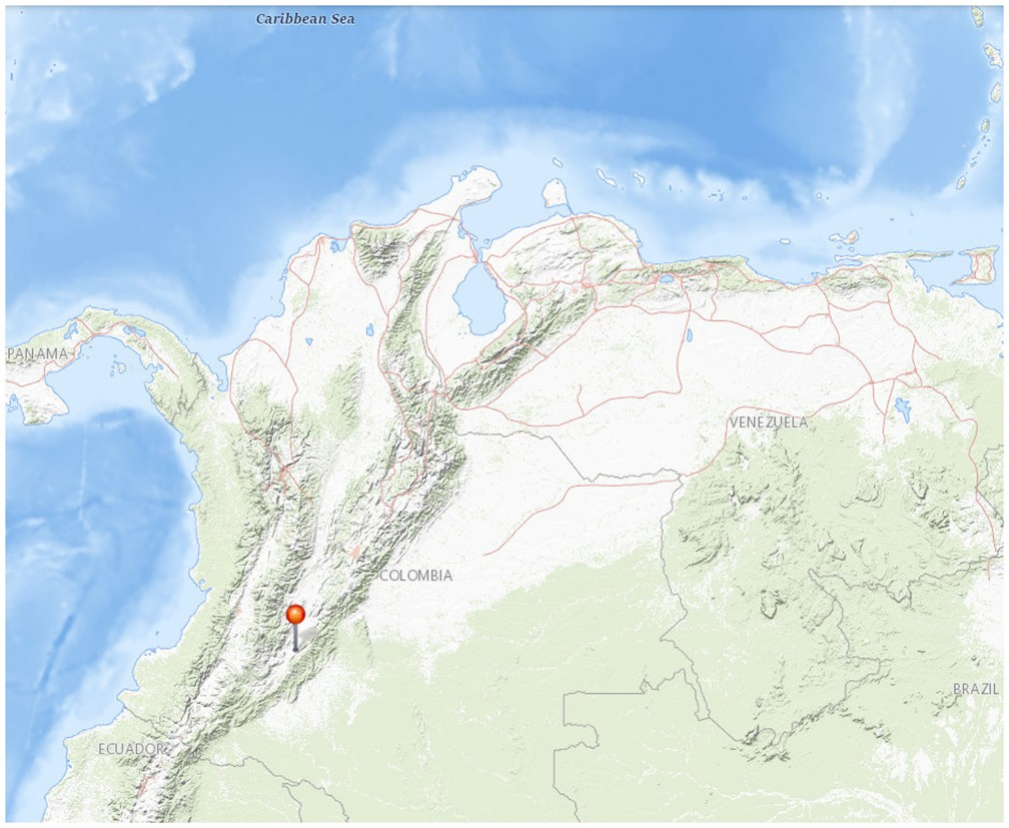
Pond variables measured every 3 hours during 48 hours. (A) Dissolved oxygen and pH. (B) Environmental and pond temperature. (C) Ammonia, ammonium and nitrites. (D) Electric conductivity and alkalinity. Note that the maximum and minimum values tend to be reached at dawn and sunset.

Fig 3 shows the measurements of the pond variables for 48 hours with a periodicity of 3 hours. As expected, Fig 3A shows the curves of pH and dissolved oxygen which are highly related to the respiration and photosynthesis cycles of the phytoplankton on the pond. At dawn, both tend to be low because algae use oxygen during the night in the respiration process, increasing the concentration of carbon dioxide in the pond. During the day, that carbon dioxide is used by the algae in the photosynthesis process, and both pH and dissolved oxygen increase, reaching a maximum peak at sunset. The presence of dissolved oxygen at dawn reflects the importance of natural water replacement because it helps naturally with the increase of dissolved oxygen in the water and avoids the need for artificial aeration. Finally, the peak of dissolved oxygen in the afternoon of the second day could be the result of an increase of phytoplankton caused by a high sun irradiance correlated with the high environmental temperature shown in Fig 3B.

Fig 3B shows that the highest temperatures in the pond and environment occur from noon to sunset having peaks at 12:00 and 18:00 hours. Although pond temperature shows a lower variance than environmental temperature, it varies more than 2°C because of the natural water replacement of the pond by the river. Fig 3C shows indicators of the nitrogen cycle in the pond for the two days of measurement. This figure does not show any relation between the feeding hour of the fish and the concentration of ammonium in the pond because both of them have a similar time periodicity (feeding and measurements). Ammonia and ammonium mass concentrations have lower variance in this pond than other more controlled ponds because the water replacement decreases the impact of feeding wastes on the parameters of the pond retiring easily the nitrogen compounds. The high variance of nitrites, mainly the highest peak, can be explained because of some wastes of other fisheries that are in the same riverbed. The curves of ammonium and nitrites show a slight inverse relation between them, mainly in afternoon hours. This result shows the oxidation process made by Nitrosomonas and Nitrobacter transforming ammonium to nitrites and after that to nitrates. Moreover, the relation of equilibrium between ammonia and ammonium in this pond is 1:15. This relation is consistent with the fraction of total ammonia existing as un-ionized ammonia showed by [5] for ponds with a temperature between 26°C and 28°C, and an average pH between 7.8 and 8. This equilibrium between ammonia and ammonium is enough to avoid any issue related to a high concentration of toxic un-ionized ammonia in the pond, which could be increased by an increase in pH or temperature of the pond. Finally, conductivity and alkalinity do not show any sharp variation at a specific hour. Only small peaks of alkalinity at the same hours mentioned for pH and dissolved oxygen, and a peak of conductivity at midnight, were observed. It is important to maintain the average alkalinity near to 90 mg/L to avoid a higher variance on the pH measurements which can impact negatively the equilibrium of ammonia and ammonium and therefore, on fish [5].

**Fig 4:**
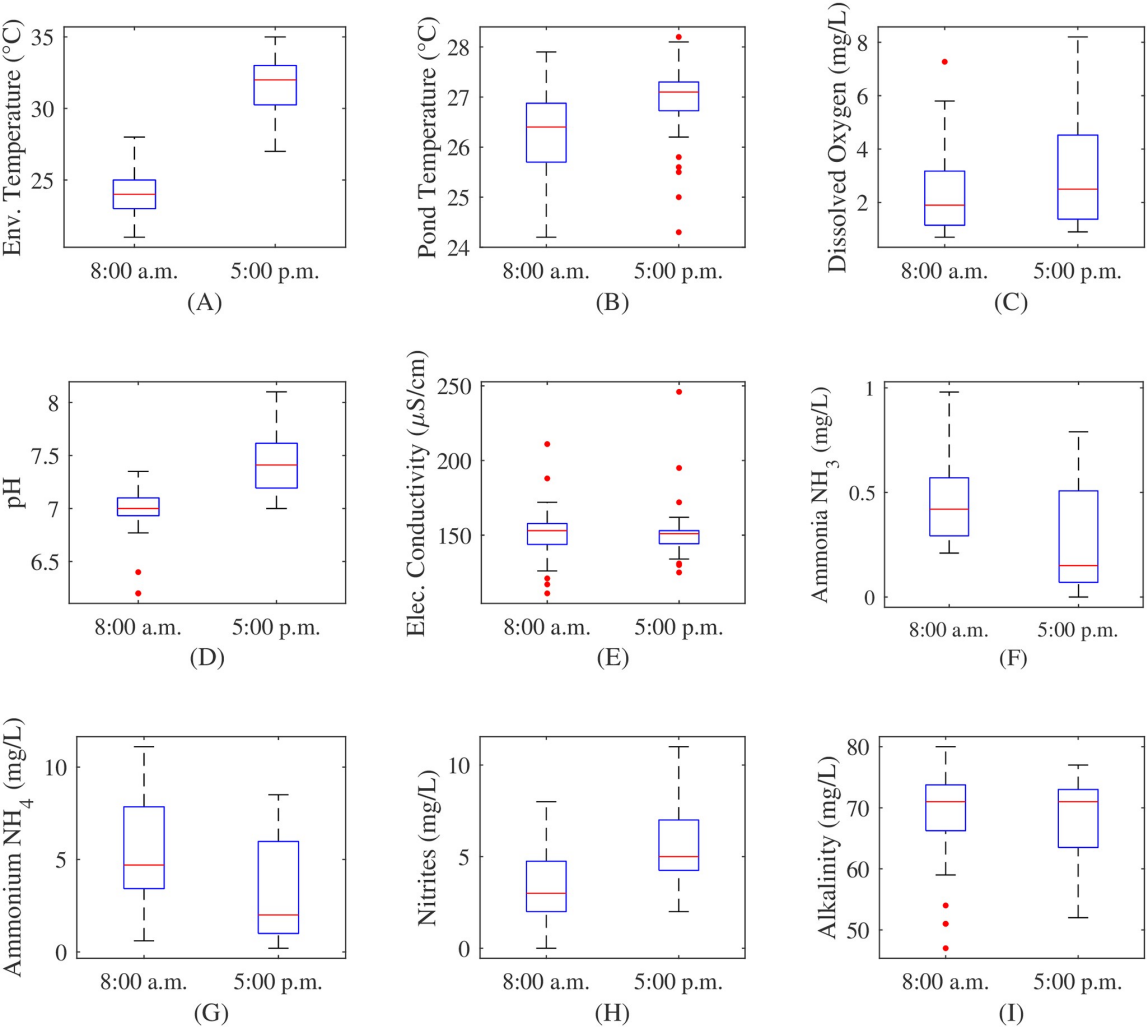
Distribution of measured variables. (A) Environmental temperature, (B) pond temperature, (C) dissolved oxygen, (D) pH, (E) electric conductivity, (F) ammonia NH_3_, (G) ammonium NH_4_, (H) nitrites, and (I) alkalinity.

These results show that measurements in this scenario should be made at dawn and sunset. Therefore, since the water pond is close to the equator, we chose to collect data at 8 a.m. and 5 p.m. during five weeks. Fig 4 shows the boxplots of all the on-site measurements at 8 a.m. and 5 p.m. Environmental and pond temperature shows lower values at dawn than at sunset. However, the difference between 8 a.m. and 5 p.m. is lower for pond temperature, showing that this temperature has not a high variation (lower than 4°C). Dissolved oxygen and pH also tend to be lower at dawn than at sunset, as it is mentioned, because of the photosynthesis and respiration cycle. Despite this difference, the deviation of dissolved oxygen measurements is high, showing that hour is not the only feature that has a high impact on this variable. Electric conductivity and alkalinity do not show a high variation between dawn and sunset measurements. Finally, variables related to the nitrogen cycle show a slight difference between measurements at 8 a.m. and 5 p.m. Ammonia and ammonium tend to be higher in the morning and nitrites tend to be higher in the afternoon. The high deviation of these variables, as it was mentioned for dissolved oxygen, allows us to hypothesize that hour is not the only important feature to estimate or forecast them.

With these data, it is possible to explore two different scenarios: variable estimation and forecasting. In addition to the water-quality variables, we collected open satellite measurements of precipitation, cloud area fraction (CAF) for low, medium, and high clouds, surface eastward and northward wind-speed, and surface specific humidity, from NASA’s MERRA-2 [18] database with the same timestamp of the on-site measurements.

### Estimation and forecasting models

A visual inspection of the behavior of the variables in Fig 3 shows that some variables are correlated. This correlation is justified by the respiration and photosynthesis cycle in the pond and the direct impact of some variables on another. For example, pH, dissolved oxygen, and pond temperature reach their maximum and minimum values at similar hours. Moreover, pH, temperature, and total ammonia determine the balance between nitrogen compounds in the pond [5]. These observations allow us to hypothesize that some variables can be estimated based on the measurements of other ones. If that is the case, variables that are accurately estimated do not require to be measured, reducing the costs of additional measurements for fish farmers. For this reason, the first approach of this study is estimation.

#### Estimation

To develop an estimation model with a limited amount of data, we selected the random forest (RF) model for regression. This model has been shown to perform well in scenarios where the amount of training data is small compared to the number of predictors [19]. Moreover, this algorithm has been used for estimation of water quality variables when the historic data of the variables to be studied is unobserved [17]. RF builds several independent decision trees applying a bagging strategy to randomly sample with replacement in the construction of each tree, reducing the variance of the result, and outputting the mean prediction of individual trees. Each decision tree splits the nodes on all available variables and then selects the split which results in the most homogeneous sub-nodes [20]. The hyperparameters of the model are the number of variables that can be split between each node, the depth of the tree, the maximum number of samples on terminal nodes, and the number of trees. An important aspect of a random forest model is that, given the trained model, it is straightforward to compute the importance of each variable to conduct the regression process [21]. To compute this importance on a trained RF model, we first calculate the importance of each node on a tree. Second, we use the importance of each node and the number of child nodes that split on each variable to find the importance of each variable on a tree. Finally, we normalize the importance of each variable on each tree and average it over all trees to calculate the variable importance of each parameter. Because of the limited number of on-site measurements, we applied 1000 runs of a validation scheme using a training set of 50 measurements and a test set of the other 20 measurements, to achieve convergence of mean and standard deviation on the performance indicators. The hyperparameters for the RF model of each variable are found using a random search. This random search is bounded between 10 and 10000 trees, and nodes of each tree are expanded until all leaves are pure.

#### Forecasting

Another important issue for fish farmers is to predict the behavior of their ponds. Therefore, the second approach of this study is forecasting. Deep learning algorithms such as recurrent and long short-term memory neural networks have been widely used in time-series forecasting [11, 13, 14]. However, unlike models such as random forests, this type of algorithm requires large datasets for training and it is difficult to extract information from the trained model for interpretation. To develop the forecasting model we used again RF and compared its results with linear regression (LR) and artificial neural network (ANN) model, which are conventional approaches to address forecasting problems. In the forecasting problem, the prediction model uses information from both other variables and historic data of the same variable. We trained a model for each on-site measured variable. For the LR model, the only hyperparameter is the regularization constant. For the ANN model, we used the multilayer perceptron topology where all neurons of a hidden layer are connected to all neurons of the following layer. We use a ReLu function as the activation function because all variables can have a value between 0 and ∞. Finally, for the three types of models, we added the hyperparameter *PO*, which corresponds to the number of previous observations that are used by the forecasting model for prediction. For example, if *PO* = 4, the model uses data collected from the last two days, that is, the last 4 observations. To train the forecasting models, we followed the same process used in the estimation approach. To train and test each model, we conducted 1000 runs of a validation scheme using 50 samples to train on each run and averaging the performance indicators of all runs. Hyperparameters are found using a random search, bounding the number of hidden layers between 1 to 5 and the neurons per layer between 2 to 16 neurons. The number of *PO* is also found in the random search algorithm bounding this hyperparameter from 0 to 6.

### Performance indicators

To measure the performance of the variable estimation and forecasting models, the following indicators are used: Mean Absolute Error (*MAE*), which measures the differences between the real and predicted values; Root Mean Square Error (*RMSE*), which quantifies the squared error in prediction, giving more weight to larger prediction mistakes; Normalized Root Mean Square Error (*NRMSE*), which normalizes the *RMSE* using the difference between the maximum and minimum value of the time series to estimate a percentage of error; and the coefficient of determination (R^2^), which estimates the variance that can be predicted with the model for each pond variable. Eqs (1) to (4) show the expressions for these indicators, with *y* and *y*_*pred*_ being the real and predicted measurements respectively, and *N* being the number of samples in the test set:

$$\begin{array}{c} MAE=\frac{1}{N}\sum |y-y_{pred}| \end{array}$$ (1)

$$\begin{array}{c} RMSE=\sqrt{\frac{1}{N}\sum {\left(y-y_{pred}\right)}^{2}} \end{array}$$ (2)

$$\begin{array}{c} NRMSE=\frac{\sqrt{\frac{1}{N}\sum {\left(y-y_{pred}\right)}^{2}}}{y_{max}-y_{min}} \end{array}$$ (3)

$$\begin{array}{c} {\text{R}}^{2}=1-\sum \frac{{\left(y-y_{pred}\right)}^{2}}{{\left(y-y_{mean}\right)}^{2}}. \end{array}$$ (4)

## Results

In this section, we first show the results of variable estimation using the random forest model, including an analysis of variable importance for estimation. Then, we present the results of variable forecasting using random forests, and compare them with linear regression and artificial neural networks. Finally, based on the feature importance for variable forecasting, we show the forecasting results with a reduced set of variables to analyze if fish farmers can forecast accurately the most important variables without requiring all measurements described in this study.

### Variable estimation

Table 1 shows the average performance indicators of the validation algorithm for measurement estimation based on other variables. This table shows that several variables cannot be estimated with high accuracy based on the collected measurements of the other variables. For example, nitrites and electric conductivity have an R^2^ lower than 0. This might occur because those variables depend on unmeasured events such as the presence of bacteria related to the nitrogen cycle or the high concentration of other ions in the pond which hinder their estimation. On the other hand, ammonium (${\text{NH}}_{4}^{+}$) and ammonia (NH_3_) are the variables that can be best estimated by the other ones. As we mentioned before, the equilibrium between ionized and un-ionized ammonia depends on pH and temperature, which are variables included in our dataset. For this reason, ammonium and ammonia can be accurately estimated. The other variables have an R^2^ between 0.3 and 0.6, which means that is possible to estimate some of their variance but is difficult to estimate them accurately.

**Table 1:** Performance indicators for measurement estimation based on other variables.

Variable	*MAE*	*RMSE*	*NRMSE* (%)	R^2^
Pond Temperature (°C)	0.4350	0.5971	0.1493	0.5501
Dissolved Oxygen (*mg*/*L*)	1.1566	1.6163	0.2155	0.3014
pH	0.1839	0.2550	0.1523	0.4059
Electric Conductivity (*μS*/*cm*)	11.512	18.989	0.2009	-0.2445
Ammonia NH_3_ (*mg*/*L*)	0.0917	0.1357	0.1385	0.6429
Ammonium ${\text{NH}}_{4}^{+}$ (*mg*/*L*)	1.1874	1.6988	0.1559	0.6753
Nitrites (*mg*/*L*)	1.8090	2.3272	0.2567	-0.0192
Alkalinity (*mg*/*L*)	3.6625	4.7386	0.1625	0.5208

Let *ϕ*_*ij*_ be the importance of variable *i* to estimate variable *j* at a given time. To quantify both the importance of a specific variable on each model and the ability of that model to estimate the target variable, we weighted the variable importance using the

$$\begin{array}{c} {\hat{\phi }}_{ij}=\phi_{ij}{\text{R}}_{j}^{2}, \end{array}$$ (5)

where ${\text{R}}_{j}^{2}$ is defined in Eq (4).

**Fig 5:**
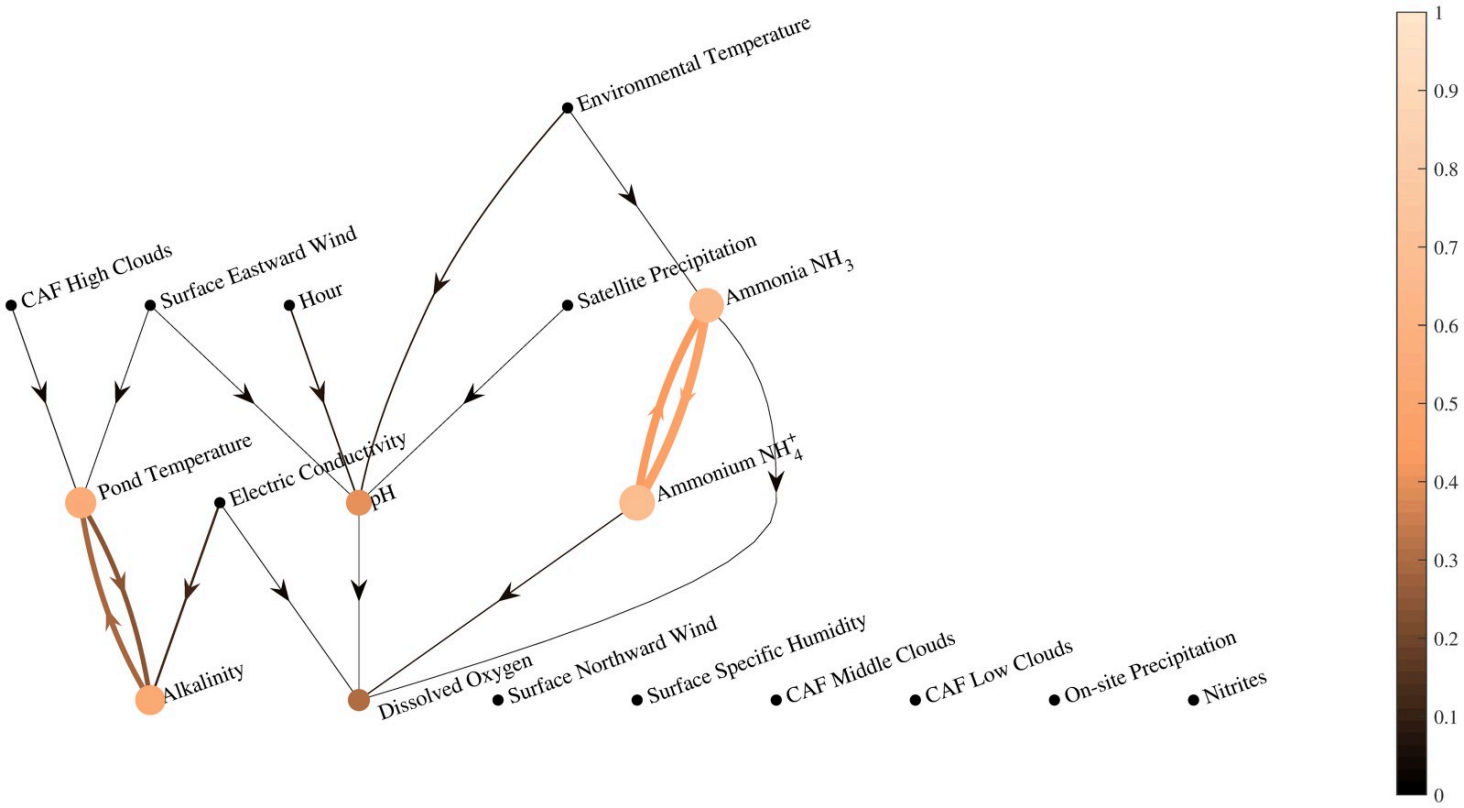
Weighted variable importance (arcs) and R^2^ (nodes) for measurement estimation based on other variables using the random forest model. Nodes with lighter colors indicate higher R^2^. Lighter colors of an arc from node *i* to node *j* indicate higher importance of variable *i* to estimate variable *j*.

Fig 5 shows the weighted variable importance for measurement estimation based on other variables and R^2^ of that estimation using the random forest algorithm. Lighter node colors indicate higher R^2^ values. Similarly, lighter colors of an arc that goes from *i* to *j* indicate higher importance of that parameter *i* to estimate variable *j*. This figure shows that the largest importance between any two variables is ionized and un-ionized ammonia as expected. This would imply that one of these measurements can potentially be discarded to reduce the costs of data collection. Other interesting relations between variables for estimation can be observed. For example, pond temperature and alkalinity have a strong relationship for estimation. Hour, precipitation, and wind-speed are useful to estimate pH, while wind-speed and clouds are important to estimate the pond temperature. Variables pH, conductivity, and both types of ammonia are important to estimate dissolved oxygen. Interestingly, none of the satellite measurements shows high importance to estimate any of the pond variables.

Furthermore, other known relations such as the data acquisition time and pH, dissolved oxygen, or pond temperature (caused by the respiration and photosynthesis cycle) were not shown in these results. A plausible explanation is that the random forest uses just one of the variables that provide that information to the model instead of using two or more variables with similar and redundant information for the estimation. For example, ammonia is not important to estimate dissolved oxygen because that information is already given to the model by the ammonium measurements. However, the low values of R^2^ for the models of pH, dissolved oxygen, and pond temperature variables indicate that, despite the relation between those variables, none of them could be estimated by just using measurements of other parameters. Table 2 summarizes the results for variable estimation.

**Table 2:** Summary of results for variable estimation.

Variable	Accurate Estimation	Important Features
Pond Temperature	Yes	Alkalinity, Wind-speed, and Clouds
Dissolved Oxygen	Medium	Ammonia, Ammonium, pH, and Electric Conductivity
pH	Medium	Hour, Environmental Temperature, Precipitation, and Wind-speed
Electric Conductivity	No	-
Ammonia	Yes	Ammonium and Environmental Temperature
Ammonium	Yes	Ammonia
Nitrites	No	-
Alkalinity	Medium	Pond Temperature and Electric Conductivity

### Variable forecasting

In this section, we evaluate the forecasting process and variable importance analysis using random forests (RF) when there are few samples for training and long forecasting horizons. We compare the results with those obtained using linear regression (LR) and artificial neural networks (ANN), which are conventional approaches to address forecasting problems. Table 3 shows the performance indicators of the RF, LR, and ANN models and the best *PO* for each variable. All methods perform similarly, being RF and LR slightly better than ANN. This can be the result of the larger amount of parameters that have to be determined by the ANN using a small dataset for training. Similar to the estimation results, the models cannot forecast electric conductivity and nitrites with high accuracy. This shows again that both parameters depend on unmeasured events such as the presence of bacteria and ions, which are difficult to estimate. Nitrites and electric conductivity are the only two parameters that have an error indicator *NRMSE* higher than 20%, showing a high performance of the models for most of the variables. Ammonia and ammonium are again the variables that can be predicted with a higher R^2^ because of the high correlation between these parameters.

**Table 3:** Performance indicators for variable forecasting. Indicator *PO* refers to the number of past observations of all variables required to forecast each variable.

Method	Variable	*MAE*	*RMSE*	*NRMSE* (%)	R^2^	*PO*
Random Forest	Pond Temperature (°C)	0.5512	0.7494	0.1874	0.2991	1
	Dissolved Oxygen (mg/L)	0.8263	1.1573	0.1543	0.6245	4
	pH	0.2085	0.2831	0.1690	0.3539	2
	Electric Conductivity (*μS*/*cm*)	11.4705	18.9025	0.2000	-0.0603	1
	Ammonia NH3 (mg/L)	0.1166	0.1816	0.1853	0.5639	4
	Ammonium NH4 (mg/L)	1.3490	1.8212	0.1671	0.6281	4
	Nitrites (mg/L)	1.8417	2.3186	0.2558	-0.0081	2
	Alkalinity (mg/L)	4.0940	5.0200	0.1721	0.3960	3
Linear Regression	Pond Temperature (°C)	0.5322	0.7032	0.1758	0.3742	1
	Dissolved Oxygen (mg/L)	0.9033	1.2383	0.1551	0.5812	4
	pH	0.2049	0.2841	0.1695	0.3610	2
	Electric Conductivity (*μS*/*cm*)	12.0685	18.2533	0.2152	-0.2973	1
	Ammonia NH3 (mg/L)	0.1198	0.1809	0.1846	0.5789	4
	Ammonium NH4 (mg/L)	1.3034	1.7659	0.1620	0.6391	4
	Nitrites (mg/L)	1.9129	2.3910	0.2674	-0.0944	2
	Alkalinity (mg/L)	4.3214	5.5530	0.1783	0.3846	3
Artificial Neural Networks	Pond Temperature (°C)	0.5957	0.7398	0.1950	0.2787	1
	Dissolved Oxygen (mg/L)	1.0065	1.3820	0.1743	0.5082	4
	pH	0.2260	0.3745	0.1871	0.1334	2
	Electric Conductivity (*μS*/*cm*)	15.9072	22.4183	0.2161	-0.1875	1
	Ammonia NH3 (mg/L)	0.1193	0.1829	0.1967	0.5332	4
	Ammonium NH4 (mg/L)	1.4216	1.9101	0.1752	0.5884	4
	Nitrites (mg/L)	1.9509	2.3744	0.2759	-0.0545	2
	Alkalinity (mg/L)	4.4405	6.5628	0.1789	0.3812	3

Models also showed to be accurate to forecast dissolved oxygen. Here, the RF and LR models showed similar and in some cases a better performance to the models presented in previous works. For example, although the models in [12] uses data that were collected every 15 minutes, most of them show a *MAE* higher than 1mg/L for dissolved oxygen for long-term forecasting (higher than 9-hour). The work in [11] forecast dissolved oxygen using different ML techniques. Similarly, all these models have a *RMSE* higher than 1.3 mg/L and an *R*^2^ lower than 0.7. Our RF and LR models have a *MAE* of 0.82 mg/L and 0.90 mg/L, a *RMSE* of 1.16 mg/L and 1.24 mg/L, and a *R*^2^ of 0.62 and 0.58. This slight improvement in forecasting can be the result of adding satellite information and on-site data related to the nitrogen cycle. However, it is important to consider that each case of study depends on the particular conditions of each water pond, and results are not directly comparable. This is clearly shown in [12] where the authors used the same methodology for two different ponds, showing a high difference in the performance indicators for each pond. Finally, as far as we know, the other variables have not been studied for long-term horizon forecasting and therefore the results are not comparable.

**Fig 6:**
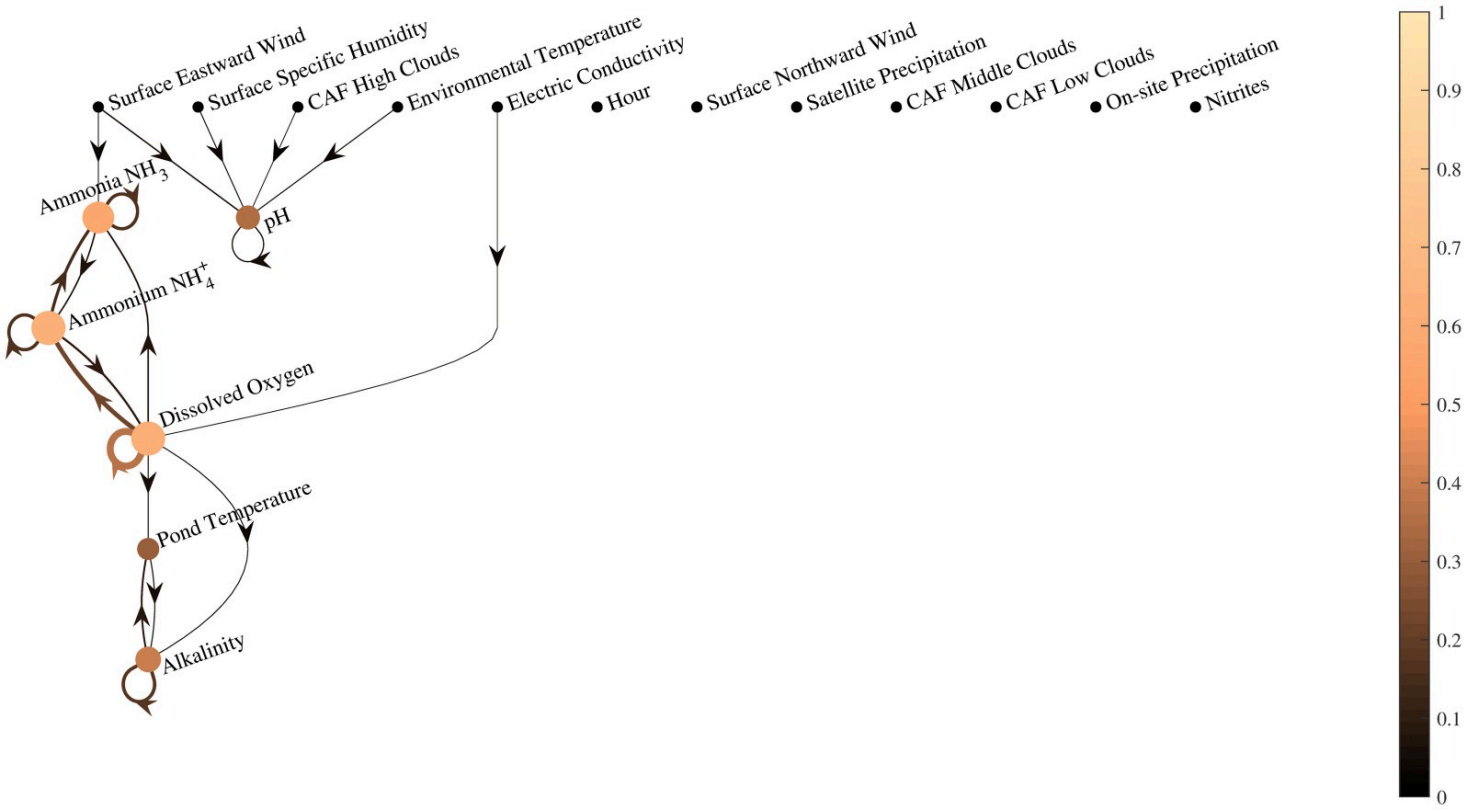
Weighted variable importance for forecasting of each parameter and R^2^ of that forecasting using the random forest algorithm. Lighter color of nodes refers to a higher R^2^. Similarly, lighter color of an arc that goes from *i* to *j* refers to a higher importance of that parameter *i* to forecast variable *j*.

Fig 6 shows the weighted variable importance for the forecasting of each parameter and the R^2^ indicator of each predictive model, using the RF algorithm. For those cases with *PO* > 1, the importance of variable *i* is computed as the sum of individual variable importance at every time step required by the model. For example, the importance of pond temperature to forecast pH (*PO* = 2) is the sum of the variable importance of pond temperature at steps *k* − 1 and *k* − 2. In this forecasting approach, previous data of the variable that is being predicted shows higher importance than any other variable. Most relations that were observed in the variable estimation process are also observed in the forecasting process, such as ionized and un-ionized ammonia, or pond temperature and alkalinity. However, the historic data of the variable to be predicted becomes more important for forecasting than measurements of the other variables. Interestingly, the importance of satellite measurements for forecasting is low, as in the estimation case. This suggests that accurate forecasting can be conducted without requiring this type of measurement in this scenario.

**Fig 7:**
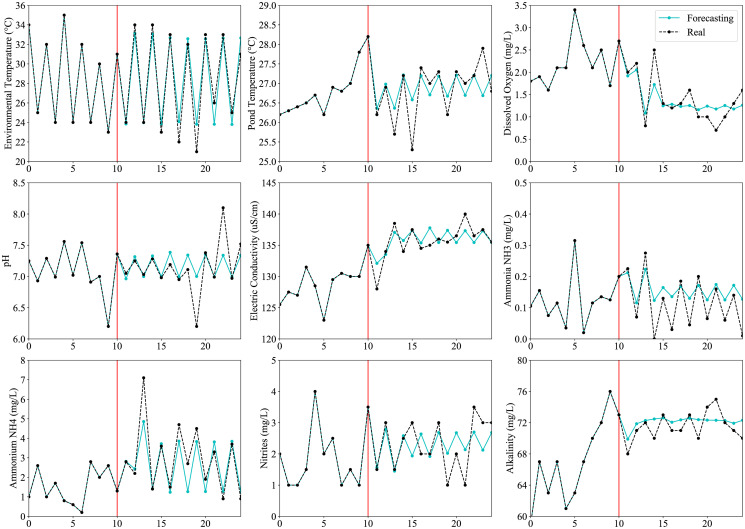
Forecasting of on-site measurements for a time horizon of 14-time steps (one week). Note that, in general, the error for the first two predicted measurements (one day) for each variable, is low.

Finally, Fig 7 compares prediction and the real measurements of each variable. This figure shows the estimated next 14 values of each variable, which is a time horizon of one week, starting from the tenth measurement. This prediction is conducted applying recursively one-step-ahead forecasting of all the models described previously. For all variables, these models show high performance predicting the first 4 measurements, demonstrating that it is possible to forecast the parameters of the water pond for a time horizon of at least one day with high accuracy. Note that when the time horizon increased, although the accuracy decreases because is harder to consider particular events that impact variables, the model tends to maintain a periodic function, changing values between morning and afternoon to minimize the error, and decreasing the difference between these two predictions (morning and afternoon) for those variables with a lower variance such as alkalinity. Table 4 summarizes the results for variable forecasting.

**Table 4:** Summary of results for variable forecasting.

Variable	Accurate Forecasting	Important Features
Pond Temperature	Medium	Dissolved Oxygen
Dissolved Oxygen	Yes	Dissolved Oxygen, Electric Conductivity, and Ammonium
pH	Medium	pH, Environmental Temperature, Clouds, Wind-speed, and Humidity
Electric Conductivity	No	-
Ammonia	Yes	Ammonia, Ammonium, Dissolved Oxygen, and Wind-speed
Ammonium	Yes	Ammonium, Dissolved Oxygen, and Ammonia
Nitrites	No	-
Alkalinity	Medium	Alkalinity, Pond Temperature, and Dissolved Oxygen

Using the results in Table 4, we reduced the input variables required for each model and conducted the training process, now focusing only on pond temperature, dissolved oxygen, pH, ammonium, and alkalinity. From this table, some variables such as electric conductivity, ammonia, and satellite measurements provide low or null information for other variable’s forecasting. We trained RF model, which was the model with the highest performance in the previous set of experiments. Table 5 shows the performance indicators of the new models that do not require all the variables measured in this work. As it is expected, when reducing the number of measurements to 5, the performance of the model decreases. However, the decrease in the performance of all models is lower than 3% in terms of *NRMSE* for all cases. This indicates that it is possible to provide fish farmers with forecasting tools that do not require excessive expenses in measurements.

**Table 5:** Performance indicators for variable forecasting based only in the most important variables using random forest algorithm.

Variable	*MAE*	*RMSE*	*NRMSE*(%)	R^2^
Pond Temperature (°C)	0.5612	0.7464	0.2140	0.2718
Dissolved Oxygen (mg/L)	0.8521	1.1787	0.1756	0.6158
pH	0.2128	0.2982	0.1897	0.3389
Ammonium NH4 (mg/L)	1.3500	1.8421	0.1816	0.6139
Alkalinity (mg/L)	4.0400	5.5044	0.2069	0.3695

## Feasibility of implementation

Both prediction and estimation can potentially be useful for fish farmers to improve their productivity. In this section, we show the performance of an information system that uses the models that were previously described and implement them in a commonly-used smart cellphone. The information system is based on HTTP protocol and Amazon Web Services (AWS). We used the tool JMeter and an Android 10 cellphone with 64 GB of storage and 4GB of RAM.

Table 6 summarizes the performance indicators of the information system. The first request that a user makes requires an estimated response time of 20 s. This request refers to the load of the historic information and the forecasting of all variables with a horizon of one week. Although this can be considered a high response time for a user, this does not affect usability given that fish farmers only require two measurements per day. Furthermore, since this first request, the information is stored in the server and is used in future requests to reduce significantly the response time. When the user loads a new set of measurements and predicts again the variables, the application only requires 451 ms to respond.

**Table 6:** Performance indicators of an information system for fish farmers implemented using amazon web services.

Performance Indicator	Value
1st Request Response Time (s)	20.97
2nd Request Response Time (s)	0.451
100-Requests Total Response Time (s)	2.337
100-Requests Average Response Time (s)	1.449
100-Requests Average Connection Time (s)	0.193
Average CPU usage (%)	3.48
Average Memory usage (MB)	95.63

To test the queue management of the server, we used 100 simultaneous requests, which is the maximum number of simultaneous requests that we believe could occur in a realistic scenario. The total response time of the application, in this case, is 2.337 s, with an average response time of 1.449 s and an average connection time to the server of 193 ms. This shows that, even in those cases where 100 users send simultaneous requests to the server, the average response time is lower to 1.5 s, with a maximum response time lower than 2.5s, which is perfectly affordable for all users. Finally, CPU and memory usage were tested for 10 minutes. During this time, the user actualizes his/her historical information of all measurements and requests forecasting multiple times. The average CPU usage is 3.48% and the average memory usage is 95.63 MB. These results show that the information system based on prediction models can be implemented in an average cellphone that fish farmers can afford without high expenses.

## Conclusions

This paper addressed the problem of training estimation and forecasting models for manually measured water quality variables in fish farming ponds when there is a limited amount of data. In our experiments, we first collected two measurements per day of water-quality variables of a fish-farming water pond. Then, we addressed the problems of variable estimation and forecasting and evaluated the importance of each feature for prediction. We found that models based on random forests can be used to estimate and forecast dissolved oxygen, pond temperature, pH, ammonia, and ammonium measuring water pond variables only twice per day. We showed that such models can be implemented in a mobile-based information system that is feasible in an average smartphone that fish farmers can afford.

We visualize this work as an important step to equip fish farmers with tools to improve their productivity, particularly in those scenarios where real-time monitoring cannot be implemented. Future research in this direction includes the analysis and development of more accurate forecasting models in scenarios where measurements are collected manually.
